# “The chameleon among diseases” - an explorative view of sarcoidosis and identification of the consequences for affected patients and relatives using qualitative interviews

**DOI:** 10.1186/s13023-023-02866-4

**Published:** 2023-09-07

**Authors:** Charlotte Hilker, Johanna Weis, Stefanie Ziehfreund, Elizabeth V. Arkema, Tilo Biedermann, Alexander Zink

**Affiliations:** 1 School of Medicine, Department of Dermatology and Allergy, Technical University of Munich, Biedersteiner Str. 29, 80802 Munich, Germany; 2 Clinical Epidemiology Division, Department of Medicine Solna, Karolinska Institutet, Stockholm, Sweden; 3 Division of Dermatology and Venereology Department of Medicine Solna, Karolinska Institutet, Stockholm, Sweden

**Keywords:** Sarcoidosis, Boeck disease, Unmet needs, Qualitative research, HrQoL, Sarcoidosis diagnostics, Sarcoidosis therapy

## Abstract

**Introduction:**

Sarcoidosis is a multisystemic disease, with the lungs being the main site of manifestation. Although the exact etiology remains unclear, both genetic and environmental factors are being discussed. Diagnostic evaluation is challenging, and the management of chronic patients and assessment of their needs proves difficult, especially in the absence of targeted therapy. Studies on sarcoidosis patients have shown that quality of life is limited even after clinically measurable parameters have resolved. The question remains how patients and their relatives perceive medical care and the diagnostic process and how these affect their well-being.

**Methods:**

Qualitative, semi-structured interviews were conducted with patients and their relatives between September 2019 and February 2020. Interviews were recorded, transcribed verbatim, and analyzed using qualitative content analysis. Deductive hypotheses were then formed based on categories according to personal aspects, symptoms, diagnostic, daily life activity, therapy, psychological aspects and wishes.

**Results:**

Fourteen patients and five relatives were included. Most patients reported subacute symptoms before the first organ-related episode. A high degree of personal initiative was required from the majority of respondents in both the diagnostic and subsequent therapeutic processes. In addition, respondents reported so-called “doctor-hopping”, a lack of specialists or contacts, and a lack of medical support. The Internet and self-help groups played a fundamental role for patients and relatives in exchanging information with other affected persons and to compensate for an existing information deficit.

**Conclusion:**

The results provide new insights into patients’ and relatives’ perceptions of the sarcoidosis diagnosis and treatment process. Identification of barriers such as a lack of physicians and an information deficit highlights potential targets for strategies to optimize sarcoidosis management.

**Supplementary Information:**

The online version contains supplementary material available at 10.1186/s13023-023-02866-4.

## Introduction

**Fig. 1:**
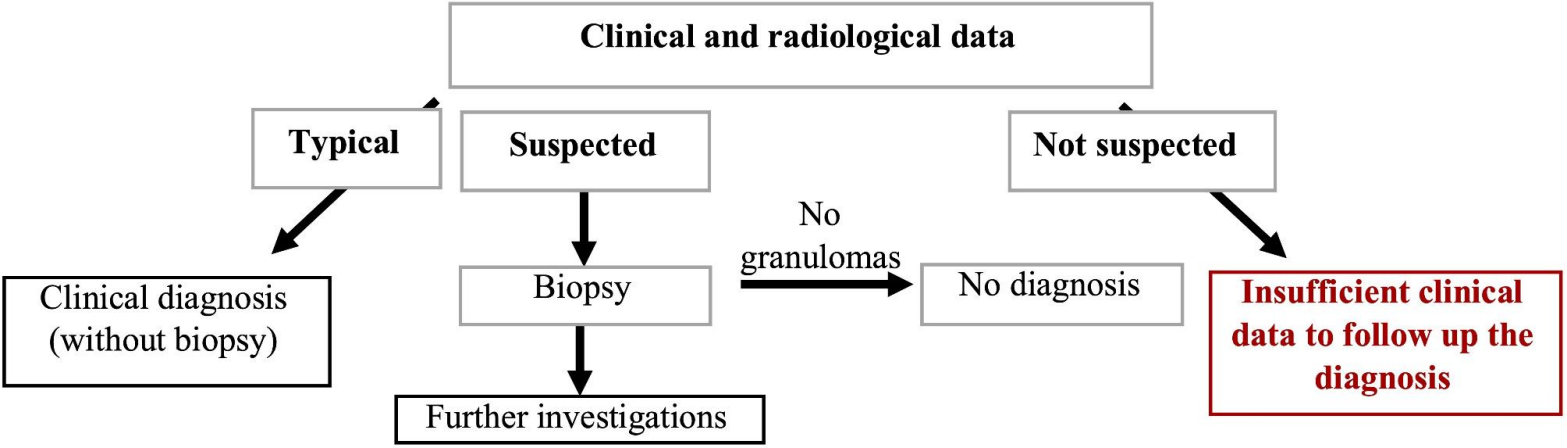
Diagnostic algorithm of sarcoidosis. Adapted by permission from Ref. ^6^, Elsevier

Sarcoidosis, also known as Boeck’s disease, is a disease of the connective tissue mostly affecting the lungs (> 90%) [1, 2]. The disease is described as the chameleon among multisystemic diseases because it varies in its manifestation, initial clinical symptoms, and course from patient to patient depending on organ involvement [1, 3]. For these reasons, diagnosis is difficult, and the diagnosis process is not standardized [1, 4, 5]. The diagnostic process of sarcoidosis predominantly takes a long time due to the atypical, heterogeneous, and non-specific clinical picture [1]. The diagnosis is made when the clinical and radiological findings are confirmed by a corresponding histology of non-caseating granulomas and if no other infectious or paraneoplastic causes can be considered (Fig. 1) [1, 6]. According to Grunewald et al., only 15% of sarcoidosis patients receive their diagnosis during their first visit to a physician [1]. Similar results were found in a Brazilian study where only 11 out of 100 patients were diagnosed during their first consultation [7]. Due to the heterogeneity of symptoms and organ involvement as well as the variable course of the disease, a comprehensive approach to care is required [8]. As the aetiology of sarcoidosis remains unknown, there are no curative treatment options, and treatment planning is challenging [1, 9]. Since the course of the disease is unpredictable and the possibility of spontaneous remission exists even in advanced sarcoidosis, it is important to weigh options between the “watch and wait” approach and drug treatment [1, 4]. Patients generally suffer from disease-related complications with reduced health-related quality of life (HRQoL) [10]. Particularly noteworthy in this context is fatigue, which is reported in up to 90% of patients and strongly associated with decreased HRQoL [11–13]. Before starting therapy, it should be discussed whether the avoidance of organ damage or the improvement of HRQoL is the primary goal, especially as pharmacological therapy can lead to numerous side effects [14–16]. The European Respiratory Society asked 1842 sarcoidosis patients to anonymously rank the most important treatment outcome parameters [17]. Quality of life and functionality were considered the two most important parameters [17]. The variability of symptomatology, the complexity of the diagnostic process, and the relevance of shared decision-making between physicians and patients regarding treatment options are demonstrated in literature [8, 15]. However, the question remains how patients and relatives perceive the diagnostic and treatment process and which influencing, and especially burdening factors affect patients’ and their relatives’ daily life.

## Materials and methods

The qualitative research was performed considering the Standards for Reporting Qualitative Research (SRQR) [18] and the Consolidated Criteria for Reporting Qualitative Research (COREQ) [19] guidelines including the Declaration of Helsinki. The study was reviewed and approved by the Ethics Committee of the Medical Faculty of the Technical University of Munich (reference: 364/19 S).

### Study subject

Patients diagnosed with sarcoidosis were recruited randomly through the German Sarcoidosis Network, the German Sarcoidosis Association, and in different clinics in Munich, Germany. To justify the theoretical saturation, people from different geographic areas and different age groups were selected using the snowball and selection procedure [18, 20, 21]. Eligible patients and relatives were required to meet the following criteria: (1) aged ≥ 18 years, (2) willing and able to provide written informed consent, (3) having a clinical diagnosis of sarcoidosis or being a relative of a patient, (4) fluent in German, and (5) no clinically diagnosed psychological disease. Interested patients and relatives were contacted by phone or mail to assess eligibility for participation, inform about the study, answer questions about the study, and arrange an appointment for a face-to-face interview. Written informed consent was obtained from all participants before any study activities.

### Data collection

A female interviewer (C.H., previous experience in conducting qualitative interviews) conducted face-to-face, semi-structured interviews in German between September 2019 and February 2020, that were audio-recorded. Most of the interviews were held at the Department of Dermatology and Allergy, Technical University of Munich. Some interviews were carried out at the participants’ homes if they were unable to attend because of the distance or their health conditions. Participants were aware of the research goals and the interviewer’s main characteristics (name, research group, and interest). The interviewer did not know any of the participants before the study. Based on the relevant literature and guidelines for conducting problem-centered interviews, an interview guide with open-ended questions was developed to ensure accurate content, clarity, and validity [20, 22–24]. The interview guide did not refer to a specific theory or model, as an exploratory character was prioritized. Nevertheless, there was an examination and reading of sarcoidosis-specific literature and the current state of research. Three pilot interviews were conducted with three healthy persons from different age groups to identify areas of potential misunderstanding and to estimate the duration of one interview [18]. The final data collection instrument consisted of three blocks, including different main and subsidiary questions.

### Analysis

All interviews were transcribed verbatim by the interviewer (C.H.). Mayring’s qualitative content analysis was used to analyze the transcripts by sorting quotes into concepts via thematic analysis methods using the qualitative data software package MAXQDA (Version: 2020.4.1) [25]. After reading the transcripts carefully to familiarize with the data, a combination of deductive and inductive formation of codes followed. Based on the structure of the interview guide, deductive codes were classified into main categories and subcategories supplemented by anchor citations for each category. New inductive categories were developed based on the data (Additional file 1: Table S1). Content saturation was judged to have been achieved if no new inductive codes were generated [25, 26]. All interviews were conducted and coded in German. The quotes reproduced below were selected for their presentation of key themes and translated into English. To achieve reliable coding, the coding structure and interrelationships were discussed by three researchers (C.H., J.W., and S.Z).

## Results

A total of 19 persons were interviewed, of whom 14 (12 women, 2 men) were patients and 5 (2 women, 3 men) relatives. Table 1 presents the characteristics of each participant. Interviews lasted between 19 and 58 min (mean 36 min). Seven categories emerged from the analysis, namely personal aspects, symptoms, diagnostics, daily life activity, therapy, psychological aspects, and wishes.

**Table 1:** Participant characteristics (patients and relatives)

Patient	Geographic area	Age Group	Sex	Organ involvement
P1	North	20–29	Female	Lung
P2	North	40–59	Female	Lung, Lymph nodes, Skin
P3	South	40–59	Female	Neurosarcoidosis
P4	South	40–59	Female	Skin
P5	South	60–80	Male	Skin
P6	South	60–80	Male	Skin
P7	South	30–39	Female	Lung, Skin
P8	South	60–80	Female	Peripheral nervous system, Lung, Lymph nodes, Eye
P9	South	40–59	Female	Eye
P10	West	40–59	Female	Neck to mediastinum, Lymph nodes, Heart
P11	North	60–80	Male	Lung
P12	South	40–59	Female	Lung, Lymph nodes, Bones
P13	South	60–80	Female	Lung
P14	South	40–59	Female	Lung, Skin, Joints
**Relative**	**Place of residence**	**Age**	**Sex**	**Relationship to associated patient**
R1	North	40–59	Male	Father to patient 1
R2	North	20–29	Female	Daughter to patient 2
R3	South	40–59	Male	Husband to patient 3
R4	South	40–59	Female	Wife to patient 5
R5	South	60–80	Male	Husband to patient 8

Patients (P) and relatives (R), North, West, East and South represent the regions in Germany where the interviewees live. Age ranges: 20–29, 30–39, 40–59, 60–80

### Personal aspects

The majority of patients and their relatives regularly inform themselves about sarcoidosis on the Internet, at congresses, in sarcoidosis networks, or through self-help groups. This self-study allowed them to improve their knowledge and become experts on the disease.

*“[…] I realized that all the physicians had never had a case like me […]. I googled a lot and asked around.“ (P10)*.

*“I’m just worried because nobody knows anything. For me as a father, that is the problem. Some of the doctors have never heard of it or don’t know how to act.“ (R1)*.

Some patients and relatives report that the information provided by the doctors was quite good. Nevertheless, almost all respondents highlighted a research and an information deficit. According to patient 10, the availability of information on sarcoidosis was “catastrophic” and P7 stated: *“I know next to nothing about the origins. Because there is not really anything for me to research in the literature. […] You must do a lot of research yourself, try to get into the networks to find the contact persons there.”*

### Diagnostic and therapy

During the diagnostic process, a high level of personal initiative was required from patients and their relatives. Participants reported that the efficiency of the diagnostic process would also depend on the engagement of individual physicians.

*“A correct diagnosis was only made by my sister-in-law. She is a pediatrician and specializes in pediatric rheumatology […]. Otherwise, we would have spent ages fiddling around with it.“ (P7)*.

*“This was a general practitioner who had studied other treatment methods intensively and he knew directly what was going on. He mentioned sarcoidosis with question marks.“ (P14)*.

Regarding the diagnostic and therapeutic process, the interviewees experienced barriers and difficulties like doctor hopping, lack of specialists, and long waiting times. One patient reported that he had first seen his general practitioner (GP), then an internist, and finally a pneumologist. Another patient was sent from doctor to doctor by her GP because of problems with her blood samples. In contrast, patient 8 reported that she was fortunately looked after by a friend of hers, who then made sure that she received an appointment with a neurologist as soon as possible.

*“The first chronic symptoms, that was a lot longer ago. It took quite a while before it was diagnosed. She had symptoms for two to three years […]. Of course, it also took a while to get an appointment with a specialist. I think waiting for the appointments always takes three to four months, which of course also delays things extremely.” (R2)*.

Among the interviewees, there were strong temporal fluctuations between the onset of the first symptoms and the final diagnosis. Eight patients received a diagnosis within three to four months after the first symptoms appeared. One patient reported that the delay between the visit to the GP and the diagnosis was one month because the pulmonologist suspected a malignancy and thereby expedited the process. A patient with sarcoidosis that affected the skin was diagnosed within two weeks. Five of the patients went through an “odyssey” lasting up to 14 years until the final diagnosis was made.

*“The first symptoms of the skin were 14 years ago, also from the lungs. […]. You were labeled a hypochondriac […]. The doctors never investigated the whole thing.“ (P14)*.

**Fig. 2:**
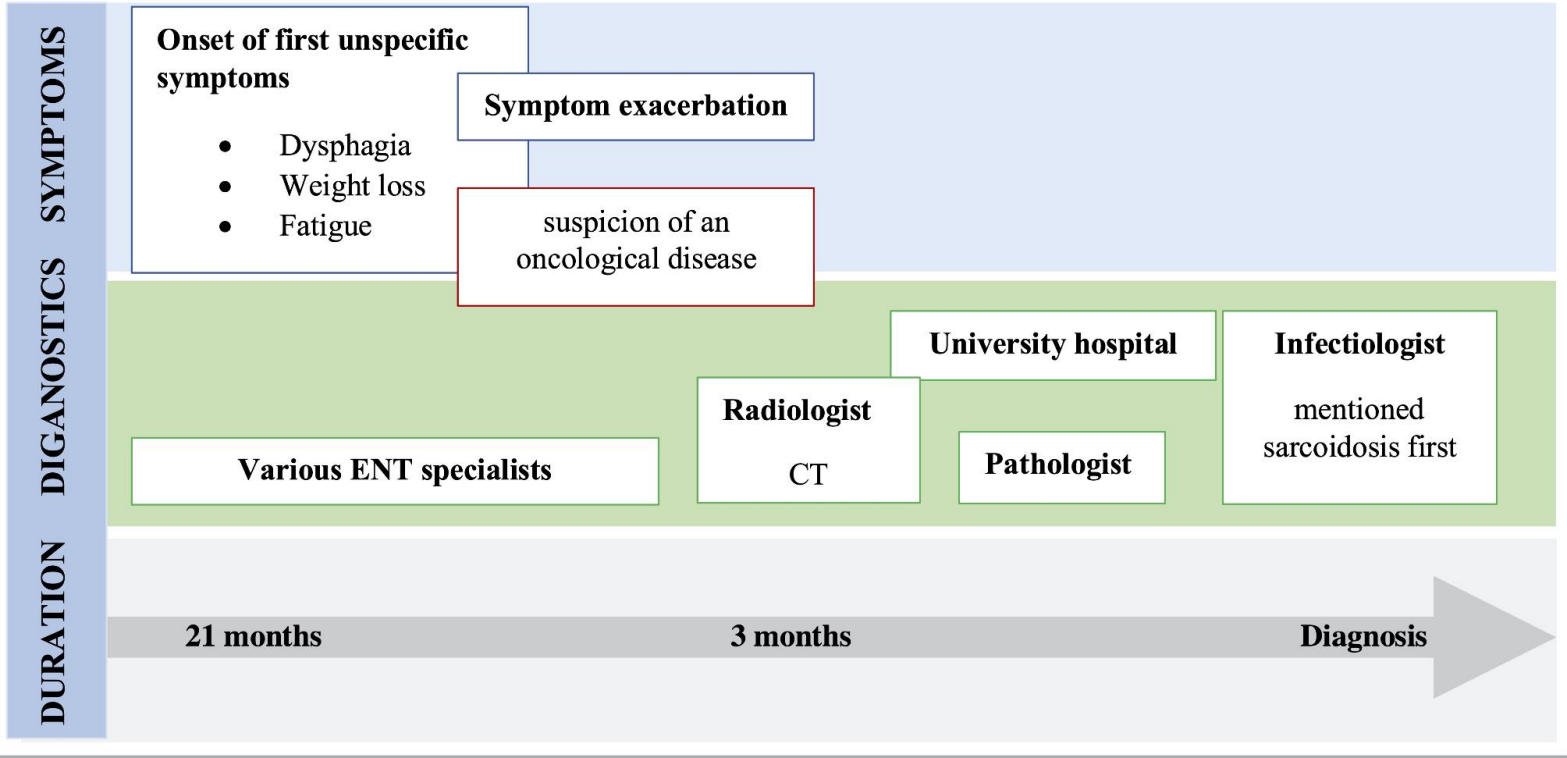
Course over time from first symptoms until diagnosis using one patient’s journey as an example. ENT; ear, nose, and throat, CT; computed tomography

The chronological course of the diagnostic process is illustrated using the patient journey of patient 10 (Fig. 2). The time delay, the changes in physicians, and the quickening of the process due to the assumption of an oncological disease being present can be observed.

*“It all went a bit back and forth […]. I think it could have been about two years, it was always up and down.“ (P10)*.

Some respondents also pointed out that they had presented to their GP with nonspecific symptoms years before the final diagnosis.

*“I had the symptoms for years before, so definitely for ten years […]. Yes, I went to the physician once, but when they come to a physician in a rural area and cough […].“ (P11)*.

Access to specialists, specialist clinics, and medical support were stated as difficult or non-existent. Another problem presented in the interviews was the lack of expertise and experience of GPs, which could delay the diagnosis and complicate the treatment process.

*“[…] I bring my general practitioner and my neurologist brochures which I get at the sarcoidosis meetings so that they can get a little informed. It was not a familiar disease to my general practitioner before.“ (P3)*.

Some of the patients and relatives felt abandoned by their disease. They connected having no guarantee of a sufficient therapy with anxiety about the future.

*“[…] I feel like I’m on my own. If you are lucky, you find someone who takes care of you and if not, you don’t.“ (P1)*.

### Daily activities

Almost all respondents reported suffering from a decreased quality of life caused by their own illness or the illness of their relatives. In particular, fatigue and reduced capacity were perceived as restrictions.

*“The biggest limitation is this accompanying rapid tiredness. The inability to cope with stress.“ (P2)*.

Depending on the organ involvement, different specific symptoms were described as impairing in everyday life. In the context of cutaneous sarcoidosis, stigmatization and associated social isolation were reported as burdening factors.

*“When it was bad, people in the underground sat away because they thought I had something contagious.“ (P4)*.

Respondents reported a reduction in activity and a loss of mobility. Some patients’ daily lives were dominated by frequent medical consultations due to the decentralized nature of healthcare. Difficulties in planning daily life due to symptom fluctuations were stated in the interviews.

*“I spend my whole life visiting the doctors […]. Basically, it’s good for me, but it bothers me in everyday life.“ (P3)*.

*“I get up early in the morning, feel good, think I can pull out trees, and then the symptoms worsen within seconds.” (P14)*.

Most of the patients were unable to organize their daily life as before, with occupational activities being reduced. One participant who is studying reported, for example, that she had applied for an academic leave for one semester. Other participants reported being on sick leave or in early retirement. Despite the physical impairment, the interviewees tried to reorient themselves professionally or looked for volunteering opportunities.

*“Eight hours of work is no longer possible because I have these fluctuations in tiredness […]. I also can’t stay up late anymore, you have your dead spot in the morning […]. I am always tired.“ (P12)*.

Some patients talked about feelings of incomprehension and rejection by their social environment. They considered one reason for this the invisible nature of sarcoidosis. Relatives recognized the social isolation and reduced participation of affected persons. Due to limited physical resources, some of these patients maintained fewer social contacts than before the onset of the disease.

*“I have also often noticed that because it is not visible, except after the surgery, it is a bit of a malingerer’s disease. “You don’t look sick at all”. Sometimes there is little understanding from other people.“ (P10)*.

*“I notice that I am making far fewer friends than I used to because I am just too knocked out.“ (P13)*.

Nevertheless, most participants experienced support from their environment. Patients experienced empathy and care from their partners. Sexual intimacy had not been affected much except during acute relapse or disease exacerbation.

*“[…] Of course he [her husband] takes care of me and spends time with me.“ (P1)*.

Self-help groups were described as important for dealing with the disease. The collection, acquisition, and exchange of information with other affected patients and their relatives were positively highlighted.

*“The exchange feels really good. Knowing that there are others who feel the same way.“ (P2)*.

All respondents agreed that the access to self-help groups was easy because of a strong online presence and availability of various networks. Several participants reported that self-help groups were performing the functions of physicians.

*“They are sometimes more competent than some doctors. They are affected people who often have more experience than the doctors.“ (P12)*.

### Wishes

The respondents expressed the desire for more professional competence, awareness, and openness in the diagnostic and therapeutic process among physicians. The disease should become more familiar, especially among GPs. Above all, the information deficit, which delays the diagnostic process, must be addressed.

*“First of all, the doctors have to become more competent. If they are competent, they would also know where to refer me or how to treat me better. But the problem is that the doctors have no idea about sarcoidosis.“ (P12)*.

Participants expressed the hope for more public attention to promote and accelerate the research process.

*“[…] More research and more international exchange. […] I don’t know why, but it’s a taboo topic. In Italy, you click once on a website, and you find sarcoidosis centers.“ (P10)*.

## Discussion

There are numerous quantitative studies on the epidemiology, clinical presentation, diagnostic options, therapies, and HRQoL of sarcoidosis. To our knowledge, this is the first qualitative study to assess the factors influencing the diagnostic and therapeutic process from patients’ and their relatives’ point of view. This study highlights the perceived reasons and consequences of the delay in diagnosis. It underlines an information deficit among physicians and the public and emphasizes the role of relatives in the acquisition of information and psychosocial support. This study also sets the scene for potential improvements and interventions in sarcoidosis health care to improve the diagnosis process and treatments to increase HRQoL among patients and relatives.

### General aspects influencing the diagnostic process and therapy

The interviews provided different indications that a deficiency of information, research, and diagnosis as well as a lack of access to specialists were perceived as burdensome by participants. Interviewed patients and relatives tackled these problems on their own by choosing online research as an approach to search for information and exchange knowledge [27, 28]. The internet offers the opportunity to assess the expertise of professionals and to compensate knowledge deficits [29, 30]. Amante et al. concluded that the demand for information via online search engines was particularly high in the cases of medical care deficits like long waiting times for specialist appointments [31]. People who have difficulties accessing health services use the internet to seek information and share knowledge about their disease in online forums [31, 32]. Babac et al. propose the establishment of a telephone service for rare diseases as one approach to solve this problem to provide high-quality and up-to-date information for patients affected by rare diseases, their relatives, and physicians [33]. The treatment of sarcoidosis is a challenge for both patients and physicians due to its unpredictable clinical course and the uncertainty about suitable treatment approaches [34]. In this context, the availability of information is of particular importance [8]. The complexity of the disease can make both knowledge transfer and physician-patient communication difficult [8, 11]. Studies have shown that patient-centered communication, which is characterized by an understanding and reassuring manner, reduces stress and improves recall of generated information such as treatment concepts [11]. Linking the knowledge of patients, relatives, and health professionals in “online patient communities” may have the potential to improve the care of chronically ill patients [32]. The interviewed subjects mentioned a lack of expertise and experience of physicians, especially among GPs, which delayed diagnosis and complicated the therapy process. GPs have an essential role to fulfill in the care of sarcoidosis patients, but they have limited prior experience and knowledge of sarcoidosis and its multiorgan character [35]. A close patient-physician relationship may improve health outcomes and facilitate collaboration with other specialists involved in the patient’s care [35, 36]. Some participants praised their GPs for playing a role in the coordination of follow-up appointments with specialists. This observation fits with findings of other studies that demonstrated how close physician-patient communication could improve health outcomes and play an important role in the care of sarcoidosis patients [11, 36].

### Rare suspicion of sarcoidosis

In general, the diagnostic process of sarcoidosis is delayed due to the complex, atypical, heterogeneous, and chameleon-like clinical picture of the disease. Non-specific symptoms such as fatigue may obstruct the diagnostic process [34, 37]. The interviews suggested that these symptoms may already be general symptoms of sarcoidosis as part of undiagnosed subacute symptoms during the onset of the disease [38, 39]. Some of the interviewed patients reported that they had presented to their GPs years in advance with symptoms. These findings suggested that sarcoidosis was rarely initially suspected, and that the diagnostic process was only immediately initiated to investigate the possibility of life-threatening diseases such as oncological diseases. Consequently, the problem may lie in the difficulty of diagnosing sarcoidosis solely using clinical parameters (Fig. 1) [6, 40]. Further laboratory, chemical, microbiological, and radiological diagnostics are only initiated if sarcoidosis is suspected [5, 6]. This corresponds to the low diagnosis rate during first consultations [7, 40]. Three of the four patients with cutaneous sarcoidosis were diagnosed within a relatively short period, which may be because of the dermatological phenotype of the disease and the homogeneous visible symptoms [37]. An additional reason could be that the dermatological patients were diagnosed in a university hospital. University hospitals offer centers for rare diseases, where patients are referred to specialists after a first assessment. Various authors have repeatedly pointed out the relevance of Sarcoidosis specialists and specialist centers [1, 17, 41, 42] as well as “the value of a multidisciplinary approach and long-term follow-up by specialized teams in sarcoidosis” [41]. Organ manifestation, physician decision-making, and patient or relative engagement may have an impact on the length of time until diagnosis. According to Grunewald et al., one needs a multidisciplinary team, increased awareness of the disease, centralized clinical care, and up-to-date guidelines [1]. This could reduce diagnostic delays and counteract the physician hopping perceived as burdensome by the participants in this study.

### Factors influencing patients’ and relatives’ daily lives

The dynamics of chronic diseases like sarcoidosis challenge patients and their relatives and affect their daily lives [6, 43, 44]. Fatigue and the decreasing ability to cope with stress were highlighted by the interviewees as limitations in daily life. The finding of previous studies that fatigue can be observed in about 50–70% of patients fits the observation that most respondents complained about similar symptomatology [38]. Fatigue is a common symptom described as “chronic post-sarcoidosis fatigue syndrome” [45], but it is not covered by diagnostic standards in clinical practice [40]. The interviewees criticized receiving inadequate care according to this symptomatology and being treated only based on laboratory parameters rather than their subjective perception of illness. Due to declining physical resilience and symptom fluctuations, affected participants described that they were no longer able to organize and plan their daily lives as before. This can be described as a negative cycle of everyday activities [11], which can cause a continuous reduction in everyday activity and consequently in HRQoL [46, 47]. Conspicuously, despite their physical limitations, patients have made efforts to participate in both their private and professional lives. The interviews also identified the restriction of occupational participation as a negative factor influencing quality of life. The findings of Hendriks et al. support this notion of affected individuals feeling as though they are not taken seriously during disability assessments [48]. Structures must be created to reintegrate affected individuals into the workforce. Attention must also be given to organ-nonspecific symptoms, such as fatigue, from which many sarcoidosis patients suffer [48, 49]. Social support is considered a salutogenetic resource of chronic diseases as “the primary place of understanding and emotional support” [50] and is a key component for stabilizing the success of treatment [30]. The interview results confirmed that family members play a fundamental role. Relatives who accompanied patients during both the diagnostic process and treatments were important for better patient care and make an effort to gain information [29, 30, 51]. Many patients experienced rejection and a lack of understanding from their social environment about their exhaustion and fatigue, especially in the acute phases of their disease. According to Moor et al., this is related to the invisible nature of the disease and may contribute to a lack of understanding of the effects of sarcoidosis, leading to social isolation and disturbed relationships [43]. Self-help groups also have an important role for the interviewees regarding the exchange of information [52]. Self-help group members develop their own competence from the exchange of experiences, particularly relating to day-to-day issues [53]. Moreover, the interviewees stated that due to the lack of contact persons, self-help groups replace medical professionals in some cases.

### Limitations

A weakness of qualitative research is that no statements can be made about frequency distributions [20, 54]. This work does not claim being able to make statements about a population and instead aims to better understand a disease in its complexity [10, 22, 55, 56]. The exploratory approach was considered suitable because to date no systematic research has focused on capturing the subjective perspective and burdening factors of sarcoidosis patients and relatives living in Germany. This study refers to the individual experiences of sarcoidosis patients and their relatives and tries to develop a deeper understanding of the biopsychosocial burdens of sarcoidosis [56]. As a next step, it would seem reasonable to examine the problems covered in this study with a quantitative study. Moreover, it should be considered that a pre-selection of participants already took place. It can be assumed that primarily those patients and relatives who were willing to talk openly about their illness participated. Bias may exist because of patients who are particularly upset about the course of their diagnosis and treatment being more likely to respond. Interviews may also be a source of social desirability bias in the study [20, 57]. Open questions and the avoidance of evaluating the statements were used to counteract this bias during interviews [58]. Most of the participants interviewed were women, with women perhaps being overrepresented in the study. However, this could also indicate that women in Germany are more frequently affected, as some studies mentioned that sarcoidosis is more common in women than in men [59]. However, other studies indicated no gender-related differences [60, 61]. It should be noted that studies have revealed gender-specific differences, with women using health services more frequently [62, 63].

## Conclusion

Clinical variability as well as the similarity to other disorders lead to a delay in sarcoidosis diagnosis and therapy. Based on the interviews, it became clear that one of the main problems appears to lie in the missing presumption of sarcoidosis during the diagnostic process. Unless sarcoidosis is suspected, no appropriate diagnosis is initiated. An interdisciplinary guideline for standardized diagnosis for different organ involvements should be developed as a principle for physicians in various disciplines. Respondents wished for more expertise and openness in the diagnostic and therapeutic process from physicians and especially from their GPs. Therefore, it is important to raise awareness of sarcoidosis in medical education. Centralization and specialization are needed to improve access to treatment options and reduce information deficits. A Germany-wide network for patients, relatives, and medical professionals such as GPs, with the assistance of a sarcoidosis platform, may improve the exchange of information.

### Electronic supplementary material

Below is the link to the electronic supplementary material.

Additional file 1: Table S1

Supplementary Material 2

## Data Availability

The datasets used and/or analyzed during the current study are available from the corresponding author on reasonable request. Since the dataset for this study are qualitative data, they cannot be made available to the public via a link or similar. Accordingly, a link or similar is not required.

## Abbreviations

COREQ: Consolidated Criteria for Reporting Qualitative Research
GP: general practitioner
HRQoL: health-related quality of life
SRQR: Reporting Qualitative Research

## References

[1] Grunewald J, Grutters JC, Arkema EV, Saketkoo LA, Moller DR (2019). Müller-Quernheim. Sarcoidosis. Nat Reviews Disease Primers.

[2] Valeyre D, Prasse A, Nunes H, Uzunhan Y, Brillet PY (2014). Müller-Quernheim. Sarcoidosis. Lancet.

[3] Tana C, Schiavone C. The Chameleon Behavior of Sarcoidosis. J Clin Med. 2021;10(13). 10.3390/jcm10132780. 10.3390/jcm10132780 PMC8269299 34202837

[4] Costabel U (2001). Sarcoidosis: clinical update. Eur Respir J.

[5] Crouser ED, Maier LA, Wilson KC, Bonham CA, Morgenthau AS, Patterson KC, Abston E, Bernstein RC, Blankstein R, Chen ES, Culver DA, Drake W, Drent M, Gerke AK, Ghobrial M, Govender P, Hamzeh N, James WE, Judson MA, Kellermeyer L, Knight S, Koth LL, Poletti V, Raman SV, Tukey MH (2020). GE Westney and RP Baughman. Diagnosis and detection of Sarcoidosis. An official american thoracic Society Clinical Practice Guideline. Am J Respir Crit Care Med.

[6] Judson MA (2008). The diagnosis of sarcoidosis. Clin Chest Med.

[7] Rodrigues MM, Coletta EN, Ferreira RG, Pereira CA (2013). Delayed diagnosis of sarcoidosis is common in Brazil. J Bras Pneumol.

[8] Moor CC, Kahlmann V, Culver DA, Wijsenbeek MS (2020). Comprehensive Care for patients with sarcoidosis. J Clin Med.

[9] Gerke AK, Judson MA, Cozier YC, Culver DA, LL Koth (2017). Disease burden and variability in sarcoidosis. Annals of the American Thoracic Society.

[10] Saketkoo LA, Russell AM, Jensen K, Mandizha J, Tavee J, Newton J, Rivera F, Howie M, Reese R, Goodman M, Hart P, Strookappe B, De Vries J, Rosenbach M, Scholand MB, Lammi MR, Elfferich M, Lower E, Baughman RP, Sweiss N, Judson MA, Drent M. Health-Related Quality of Life (HRQoL) in sarcoidosis: diagnosis, management, and Health Outcomes. Diagnostics (Basel). 2021;11(6). 10.3390/diagnostics11061089. 10.3390/diagnostics11061089 PMC8232334 34203584

[11] Drent M, Strookappe B, Hoitsma E, De Vries J. Consequences of Sarcoidosis. *Clin Chest Med*. 2015;36 (4): 727 – 37. 10.1016/j.ccm.2015.08.013. 10.1016/j.ccm.2015.08.013 26593145

[12] Michielsen HJ, Drent M, Peros-Golubicic T, De Vries J (2006). Fatigue is associated with quality of life in sarcoidosis patients. Chest.

[13] Voortman M, Hendriks CMR, Elfferich MDP, Bonella F, Møller J, Vries JD, Costabel U, Drent M (2019). The burden of sarcoidosis symptoms from a patient perspective. Lung.

[14] James WE, Baughman R (2018). Treatment of sarcoidosis: grading the evidence. Expert Rev Clin Pharmacol.

[15] Baughman RP, Valeyre D, Korsten P, Mathioudakis AG, Wuyts WA, Wells A, Rottoli P, Nunes H, Lower EE, Judson MA, Israel-Biet D, Grutters JC, Drent M, Culver DA, Bonella F, Antoniou K, Martone F, Quadder B, Spitzer G, Nagavci B, Tonia T. Rigau and DR Ouellette. ERS clinical practice guidelines on treatment of sarcoidosis. Eur Respir J. 2021;58(6). 10.1183/13993003.04079-2020. 10.1183/13993003.04079-2020 34140301

[16] Obi ON (2020). Health-Related Quality of Life in Sarcoidosis. Semin Respir Crit Care Med.

[17] Baughman RP, Barriuso R, Beyer K, Boyd J, Hochreiter J, Knoet C, Martone F, Quadder B, Richardson J, Spitzer G, Valeyre D, Ziosi G. Sarcoidosis: patient treatment priorities. ERJ Open Res. 2018;4(4). 10.1183/23120541.00141-2018. 10.1183/23120541.00141-2018 PMC6302206 30588477

[18] BC O’Brien IB, Harris TJ, Beckman DA, Reed, Cook DA (2014). Standards for reporting qualitative research: a synthesis of recommendations. Acad Med.

[19] Tong A, Sainsbury P, Craig J (2007). Consolidated criteria for reporting qualitative research (COREQ): a 32-item checklist for interviews and focus groups. Int J Qual Health Care.

[20] Meyen M, Löblich M, Pfaff-Rüdiger S, Riesmeyer C (2019). Vom Alltag ins Feld. Qualitative Forschung in der Kommunikationswissenschaft.

[21] Fuchs-Heinritz W. Biographische Forschung: Eine Einführung in Praxis und Methoden. edn.: Springer-Verlag; 2015.

[22] Meyen M, Löblich M, Pfaff-Rüdiger S, Riesmeyer C. Wie man das „richtige lager findet und Qualität sichert: Dimensionen und Gütekriterien qualitativer Forschung. Qualitative Forschung in der Kommunikationswissenschaft. Springer; 2019. 21–45.

[23] Helfferich C. Interviewplanung und intervieworganisation. Die Qualität qualitativer Daten. Springer; 2009. 167–93.

[24] Helfferich C. Leitfaden-und Experteninterviews. Handbuch Methoden der empirischen Sozialforschung. Springer; 2019. 669–86.

[25] Mayring P, Fenzl T. Qualitative inhaltsanalyse. Handbuch Methoden der empirischen Sozialforschung. Springer; 2019. 633–48.

[26] Meyen M, Löblich M, Pfaff-Rüdiger S. and C Riesmeyer. Auswertung und Forschungsbericht Qualitative Forschung in der Kommunikationswissenschaft. Springer 2019. 169–94.

[27] Hilker C, Tizek L, Rüth M, Schielein M, Biedermann T, Zink A (2021). Leveraging internet search data to assess prevalence, interest, and unmet needs of sarcoidosis in Germany. Sci Rep.

[28] Seidl S, Schuster B, Ruth M, Biedermann T, Zink A (2018). What do Germans want to know about skin Cancer? A nationwide Google search analysis from 2013 to 2017. J Med Internet Res.

[29] Haslbeck J, Klein M, Bischofberger I, Sottas B (2015). Leben mit chronischer Krankheit. Die Perspektive von Patientinnen, Patienten und Angehörigen. Obsan Dossier.

[30] Schöneberger C, von Kardorff E. Angehörige chronisch kranker Menschen. Mit dem kranken Partner leben. edn. Wiesbaden VS Verlag für Sozialwissenschaften; 2004.

[31] Amante DJ, Hogan TP, Pagoto SL, English TM, Lapane KL (2015). Access to care and use of the internet to search for health information: results from the US National Health interview survey. J Med Internet Res.

[32] Fox S (2013). After Dr Google: peer-to-peer health care. Pediatrics.

[33] Babac A, Frank M, Pauer F, Litzkendorf S, Rosenfeldt D, Luhrs V, Biehl L, Hartz T, Storf H, Schauer F, Wagner TOF, JM Graf von der Schulenburg (2018). Telephone health services in the field of rare diseases: a qualitative interview study examining the needs of patients, relatives, and health care professionals in Germany. BMC Health Serv Res.

[34] Drent M, Crouser ED, Grunewald J (2021). Challenges of Sarcoidosis and its management. N Engl J Med.

[35] Judson MA (2007). The management of sarcoidosis by the primary care physician. Am J Med.

[36] Aladesanmi OA (2004). Sarcoidosis: an update for the primary care physician. MedGenMed.

[37] Jeny F, Bernaudin J-F, Aubart FC, Brillet P-Y, Bouvry D, Nunes H, Valeyre D (2020). Diagnosis issues in sarcoidosis. Respiratory Med Res.

[38] Drent M, Lower EE, De Vries J (2012). Sarcoidosis-associated fatigue. Eur Respir J.

[39] Kidd DP (2020). Neurosarcoidosis: clinical manifestations, investigation and treatment. Pract Neurol.

[40] Grunewald J, Grutters JC, Arkema EV, Saketkoo LA, Moller DR, Müller-Quernheim J (2019). Sarcoidosis (primer). Nat Reviews: Disease Primers.

[41] Mañá J, Rubio-Rivas M, Villalba N, Marcoval J, Iriarte A, Molina-Molina M, Llatjos R, García O, Martínez-Yélamos S, Vicens-Zygmunt V, Gámez C, Pujol R, Corbella X (2017). Multidisciplinary approach and long-term follow-up in a series of 640 consecutive patients with sarcoidosis: Cohort study of a 40-year clinical experience at a tertiary referral center in Barcelona, Spain. Med (Baltim).

[42] Valeyre D, Jeny F, Rotenberg C, Bouvry D, Uzunhan Y, Sève P, Nunes H, Bernaudin JF (2021). How to tackle the diagnosis and treatment in the diverse scenarios of Extrapulmonary Sarcoidosis. Adv Ther.

[43] Moor C, Van Manen M, van Hagen P, Miedema J, van den Toorn L, Gür-Demirel Y, Berendse A, van Laar J, Wijsenbeek M (2018). Needs, perceptions and education in sarcoidosis: a live interactive survey of patients and partners. Lung.

[44] D Schaeffer (2006). Managing chronic illness implications for the health care system. Z Gerontol Geriatr.

[45] Korenromp IH, Grutters JC, Van den Bosch JM, Heijnen CJ (2012). Post-inflammatory fatigue in sarcoidosis: personality profiles, psychological symptoms and stress hormones. J Psychosom Res.

[46] MA Judson (2015). Quality of life assessment in sarcoidosis. Clin Chest Med.

[47] Marcellis RG, Lenssen AF, de Vries J, Drent M (2013). Reduced muscle strength, exercise intolerance and disabling symptoms in sarcoidosis. Curr Opin Pulm Med.

[48] Hendriks CMR, Saketkoo LA, Elfferich MDP, Vries JD, Wijnen P, Drent M (2019). Sarcoidosis and work participation: the need to develop a Disease-Specific Core Set for Assessment of Work ability. Lung.

[49] Arkema EV, Eklund A, Grunewald J, Bruze G (2018). Work ability before and after sarcoidosis diagnosis in Sweden. Respir Med.

[50] Haslbeck J, Klein M, Bischofberger I, Sottas B. Leben mit chronischer Krankheit: Die Perspektive von Patientinnen, Patienten und Angehörigen. https://formative-works.ch/wp-content/uploads/2020/01/2015_9_obsan_dossier_46.pdf. Accessed 20 April 2023.

[51] Årestedt L, Persson C, Benzein E (2014). Living as a family in the midst of chronic illness. Scand J Caring Sci.

[52] Hundertmark-Mayser J, Helms U (2019). Self-help support for chronic illness-societal challenges and current approaches. Bundesgesundheitsblatt Gesundheitsforschung Gesundheitsschutz.

[53] Danner M, Schmacke N (2019). Patient involvement: challenges for organized self-help and joint self-government. Bundesgesundheitsblatt Gesundheitsforschung Gesundheitsschutz.

[54] Pyo J, Lee W, Choi EY, Jang SG, Ock M (2023). Qualitative research in Healthcare: necessity and characteristics. J Prev Med Public Health.

[55] Cleland, J. A. (2017). The qualitative orientation in medical education research. Korean J Med Educ.

[56] Saketkoo LA, Pauling JD (2018). Qualitative methods to Advance Care, diagnosis, and Therapy in Rheumatic Diseases. Rheum Dis Clin North Am.

[57] Bergen N (2020). Everything is perfect, and we have no problems: detecting and limiting Social Desirability Bias in qualitative research. Qual Health Res.

[58] Meyen M, Löblich M, Pfaff-Rüdiger S, Riesmeyer C. Befragung. Qualitative Forschung in der Kommunikationswissenschaft. Springer; 2019. 77–112.

[59] Rybicki BA, Major M, Maliank JP, MC lannuzzi (1997). Racial differences in sarcoidosis incidence: a 5-year study in a health maintenance organization. Am J Epidemiol.

[60] Arkema EV, Grunewald J, Kullberg S, Eklund A, Askling J (2016). Sarcoidosis incidence and prevalence: a nationwide register-based assessment in Sweden. Eur Respir J.

[61] Ungprasert P, Crowson CS, Matteson EL (2017). Influence of gender on Epidemiology and Clinical Manifestations of Sarcoidosis: a Population-Based Retrospective Cohort Study 1976–2013. Lung.

[62] Bertakis KD, Azari R, Helms LJ, Callahan EJ, Robbins JA (2000). Gender differences in the utilization of health care services. J Fam Pract.

[63] Redondo-Sendino Á, Guallar-Castillón P, Banegas JR, Rodríguez-Artalejo F (2006). Gender differences in the utilization of health-care services among the older adult population of Spain. BMC Public Health.

