# Supplementary material for: “The chameleon among diseases” - an explorative view of sarcoidosis and identification of the consequences for affected patients and relatives using qualitative interviews
Source: Orphanet J Rare Dis. 2023 Sep 7;18:276. doi: 10.1186/s13023-023-02866-4 (PMC10486085; doi:10.1186/s13023-023-02866-4)
Supplement: Supplementary file 1 — Additional file 1: Table S1 [file 13023_2023_2866_MOESM1_ESM.docx]

**Table 1S**: The category system

| **Category** | **Subcategory** |
| --- | --- |
| Personal Aspects | Age  Occupation  Work  Family history  Disease history  Knowledge of disease |
| Symptoms | Organ involvement  Initial symptoms  Intensity  Intervening factors  Everyday burden  Symptom overview |
| Diagnostics | Diagnostic process  Examinations  Duration  Follow-up diagnostics |
| Daily life activity | Stressful factors and anxiety  Daily life  Social life  Partnership |
| Therapy | Pharmacological therapy  Outcome  Side effects  Specialists/Centres  General practitioner  Alternative therapy  Study participation |
| Psychological aspects | Psychotherapy  Unknown aetiology  Self-help groups |
| Wishes | Wishes |
