# Supplementary material for: “The chameleon among diseases” - an explorative view of sarcoidosis and identification of the consequences for affected patients and relatives using qualitative interviews
Source: Orphanet J Rare Dis. 2023 Sep 7;18:276. doi: 10.1186/s13023-023-02866-4 (PMC10486085; doi:10.1186/s13023-023-02866-4)
Supplement: Supplementary file 2 — Supplementary Material 2 [file 13023_2023_2866_MOESM2_ESM.docx]

Klinik und Poliklinik

für Dermatologie und Allergologie am Biederstein

des Klinikums rechts der Isar

der Technischen Universität München

Anstalt des öffentlichen Rechts

Direktor: Univ.-Prof. Dr. med. Tilo Biedermann

Biedersteiner Strasse 29, 80802 Munich, Germany

Address:	Biedersteiner strasse 29

80802 Munich, Germany

Telephone:	0049 89 41 40-3170

E-Mail:	alexander.zink@tum.de

Prof. Dr. med. Cristina Has

Editorial Board Member

Orphanet Journal of Rare Diseases

March 20^th^, 2023

**Submission ID OJRD-D-22-00616**

**“The chameleon among diseases”- An explorative view of sarcoidosis and identification of the consequences for affected patients and relatives using qualitative interviews**

Dear Mrs. Has,

Thank you for your kind and favorable reply. We highly appreciate your time and effort considering our manuscript for publication and are delighted to submit a revised manuscript of our study titled “The chameleon among diseases - An explorative view of sarcoidosis and identification of the consequences for affected patients and relatives using qualitative interviews”.

According to your and the reviewer’s suggestions, we have made several changes to the revised manuscript which are meticulously described on the next pages (point-to-point-response) and highlighted in red color in the attached document.

We are very thankful for all comments which we believe have substantially improved our manuscript. Please feel free to contact us at any time at your convenience and let us know, if we can further improve our manuscript in order to be published in the Orphanet Journal of Rare Diseases.

Thank you for your consideration. We hope our manuscript is suitable for publication in your journal.

Yours sincerely,

Dr. Alexander Zink

on behalf of all authors

Reviewer #1:

Dear reviewers,

We appreciate your thoughtful comments. We have revised our manuscript substantially to reflect your suggestions. Please see our point-by-point response to the reviewers. All changes in the manuscript have been highlighted in red. We hope you will find our manuscript suitable for publication as an Original in Orphanet Journal of Rare Diseases.

The authors describe a major problem of medical care in the field of rare diseases using the example of sarcoidosis. In doing so, they use a publication variant that is atypical for most medical journals, namely qualitative interviews. Multiple personal opinions are cited without frequency distributions in a small cohort of sarcoidosis patients who have a disease manifestation in different organs (lung > skin > lymph nodes, etc.). In my opinion, the case numbers are too small and not very representative as already mentioned in the limitations.

***Author response:*** *The exploratory approach of our study was chosen since to date no systematic research has focused on capturing the subjective perspective and burdening factors of sarcoidosis patients and relatives living in Germany. Qualitative research is used to identify, describe and explain subjective phenomena (Lamnek & Krell, 2016). It “provides valuable insight into patient experiences, attitudes, behavior, meaning, thoughts and broadens our understanding of human disease, and thusly in recent years, has carved a bona fide position as valuable science in healthcare operations and understanding of disease” (Saketkoo & Pauling, 2018). Since this study refers to the individual experiences of sarcoidosis patients and their relatives and tries to develop a deeper understanding of the biopsychosocial burdens of sarcoidosis, an explorative approach appeared to be appropriate (Pyo, Lee, Choi, Jang, & Ock, 2023; Saketkoo & Pauling, 2018; Saketkoo et al., 2021). This work does not claim to be able to make statements about a population but aims to better understand a disease in its complexity* *by looking at different perspectives and experiences (Pyo et al., 2023). As a next step, it would seem reasonable to examine the issues covered in this study with a quantitative study. During the evaluation, the principle of content saturation was applied; this was present during coding as soon as no more new inductive codes could be generated (Meyen, Löblich, Pfaff-Rüdiger, & Riesmeyer, 2019; Vogt & Werner, 2014). In the evaluation, a theoretical saturation could be achieved in large parts; in some categories, such as the diagnostic process, further interviews could have generated more insights. However, this also provides insight into the fact that the diagnostic process is largely non-standardized.*

Here, one can refer in parallel to the publications of CHERH - Center for Health Economics Research Hannover (e.g., PMID 29426339, 32503483), in which the authors describe how patients with rare diseases can get better and faster access to high quality information and thus support diagnosis finding.

***Author response:*** *Thank you for this comment and the suggested research. We referred in parallel to the publications of CHERH - Center for Health Economics Research Hannover. The establishment of a telephone service as one possible solution for the information need of patients, relatives as well as physicians. Since the Internet was repeatedly mentioned in the interviews as a resource due to the lack of contact points, a telephone service appears to be particularly useful since it is low-threshold. We have added the following sentence in our discussion: “Babac et al. propose the establishment of a telephone service for rare diseases as one approach to solve this problem to provide high-quality and up-to-date information for patients affected by rare diseases, their relatives, and physicians (Babac et al., 2018).”*

In addition, the benefits, and structures of rare disease centers in various university hospitals should be briefly described, as well as the role of central coordinators and trained staff to refer patients to the appropriate specialists. The authors could also offer further constructive suggestions on how other rare disease fields have promoted specialization and training. If there are data on how long it takes to be diagnosed with sarcoidosis, please cite population studies or publications.

***Author response:*** *Thank you for this comment and the suggested additional information. We share the opinion that specialty centers as for example in university hospitals play an important role in the diagnostic and therapy process. Various authors have repeatedly pointed out the relevance of Sarcoidosis specialists and specialist centers (Baughman et al., 2018; Grunewald et al., 2019; Mañá et al., 2017; Valeyre et al., 2021) as well as “the value of a multidisciplinary approach and long-term follow-up by specialized teams in sarcoidosis”(Mañá et al., 2017). Especially with regard of the rarer manifestations such as neurological sarcoidosis (Baughman et al., 2018). Therefore, we have added the following to our discussion: “An additional reason could be that the dermatological patients were diagnosed in a university hospital. University hospitals offer centers for rare diseases, where patients are referred to specialists after a first assessment. Various authors have repeatedly pointed out the relevance of Sarcoidosis specialists and specialist centers (Baughman et al., 2018; Grunewald et al., 2019; Mañá et al., 2017) as well as “the value of a multidisciplinary approach and long-term follow-up by specialized teams in sarcoidosis”(Mañá et al., 2017).”*

To improve care, recommend the development of an interdisciplinary guideline for standardized diagnosis for all possible organ system involvement.

***Author response:*** *Thank you for this comment. We agree on the recommendation of an interdisciplinary guideline for standardized diagnosis for different possible involved organ system. That is why we added the following sentence to our conclusion: “An interdisciplinary guideline for standardized diagnosis for different organ involvements should be developed as a principle for physicians in various disciplines.”*

Reviewer #2:

This is an interesting analysis on a rare multisystemic disease, sarcoidosis. Its often unspecific clinical findings and involvement of various organs make the diagnosis difficult. However the diagnosis should be possible in an academic hospital. Patients with unclear diagnosis should present to university hospitals and to centers for rare diseases. Such centers are now established in many universities in Germany. It was part of the national plan for rare disease in Germany to improve medical care for people living with rare diseases. Better dissemination of information regarding centers for rare diseases should replace patients google of disease symptoms.

***Author response:*** *Thank you for you comment. We totally agree that university hospitals and specialized centers can play an important role in the diagnosis and treatment of sarcoidosis. Therefore, we have added the following to our discussion: “An additional reason could be that the dermatological patients were diagnosed in a university hospital. University hospitals offer centers for rare diseases, where patients are referred to specialists after a first assessment. Various authors have repeatedly pointed out the relevance of Sarcoidosis specialists and specialist centers (Baughman et al., 2018; Grunewald et al., 2019; Mañá et al., 2017; Valeyre et al., 2021) as well as “the value of a multidisciplinary approach and long-term follow-up by specialized teams in sarcoidosis”(Mañá et al., 2017).”*

Babac, A., Frank, M., Pauer, F., Litzkendorf, S., Rosenfeldt, D., Lührs, V., . . . Graf von der Schulenburg, J. M. (2018). Telephone health services in the field of rare diseases: a qualitative interview study examining the needs of patients, relatives, and health care professionals in Germany. *BMC Health Serv Res, 18*(1), 99. doi:10.1186/s12913-018-2872-9

Baughman, R. P., Barriuso, R., Beyer, K., Boyd, J., Hochreiter, J., Knoet, C., . . . Ziosi, G. (2018). Sarcoidosis: patient treatment priorities. *ERJ Open Res, 4*(4). doi:10.1183/23120541.00141-2018

Grunewald, J., Grutters, J. C., Arkema, E. V., Saketkoo, L. A., Moller, D. R., & Müller-Quernheim, J. (2019). Sarcoidosis. *Nature Reviews Disease Primers, 5*(1), 45. doi:10.1038/s41572-019-0096-x

Lamnek, S., & Krell, C. (2016). Qualitative Sozialforschung: mit Online-Material, 6., überarbeitete Auflage. ed. Beltz. In: Weinheim Basel.

Mañá, J., Rubio-Rivas, M., Villalba, N., Marcoval, J., Iriarte, A., Molina-Molina, M., . . . Corbella, X. (2017). Multidisciplinary approach and long-term follow-up in a series of 640 consecutive patients with sarcoidosis: Cohort study of a 40-year clinical experience at a tertiary referral center in Barcelona, Spain. *Medicine (Baltimore), 96*(29), e7595. doi:10.1097/md.0000000000007595

Meyen, M., Löblich, M., Pfaff-Rüdiger, S., & Riesmeyer, C. (2019). Auswertung und Forschungsbericht. In *Qualitative Forschung in der Kommunikationswissenschaft*. Springer.

Pyo, J., Lee, W., Choi, E. Y., Jang, S. G., & Ock, M. (2023). Qualitative Research in Healthcare: Necessity and Characteristics. *J Prev Med Public Health, 56*(1), 12-20. doi:10.3961/jpmph.22.451

Saketkoo, L. A., & Pauling, J. D. (2018). Qualitative Methods to Advance Care, Diagnosis, and Therapy in Rheumatic Diseases. *Rheum Dis Clin North Am, 44*(2), 267-284. doi:10.1016/j.rdc.2018.01.004

Saketkoo, L. A., Russell, A. M., Jensen, K., Mandizha, J., Tavee, J., Newton, J., . . . Drent, M. (2021). Health-Related Quality of Life (HRQoL) in Sarcoidosis: Diagnosis, Management, and Health Outcomes. *Diagnostics (Basel), 11*(6). doi:10.3390/diagnostics11061089

Valeyre, D., Jeny, F., Rotenberg, C., Bouvry, D., Uzunhan, Y., Sève, P., . . . Bernaudin, J. F. (2021). How to Tackle the Diagnosis and Treatment in the Diverse Scenarios of Extrapulmonary Sarcoidosis. *Adv Ther, 38*(9), 4605-4627. doi:10.1007/s12325-021-01832-5

Vogt, S., & Werner, M. (2014). Forschen mit Leitfadeninterviews und qualitativer Inhaltsanalyse. *URL:* [*https://www*](https://www)*. f01. th-koeln. de/imperia/md/content/sozialearbeitplus/skript_interviewsqual_inhaltsanalyse. pdf (Stand: 09.11. 2015)*.
